# Supplementary material for: Antimicrobial resistance, virulence gene profiles, and molecular epidemiology of enterococcal isolates from patients with urinary tract infections in Shanghai, China
Source: Microbiol Spectr. 2024 Nov 29;13(1):e01217-24. doi: 10.1128/spectrum.01217-24 (PMC11705914; doi:10.1128/spectrum.01217-24)

**Table S1**: The Primers used for MLST typing of housekeep genes in *Enterococcus faecalis*

| Gene | Primer Name | Primer Sequence (5' → 3') |
| --- | --- | --- |
| *gdh* | *gdh* -1 | GGCGCACTAAAAGATATGGT |
|  | *gdh* -2 | CCAAGATTGGGCAACTTCGTCCCA |
| *gyd* | *gyd*-1 | CAAACTGCTTAGCTCCAATGGC |
|  | *gyd*-2 | CATTTCGTTGTCATACCAAGC |
| *pstS* | *pstS* -1 | CGGAACAGGACTTTCGC |
|  | *pstS* -2 | ATTTACATCACGTTCTACTTGC |
| *gki* | *gki*-1 | GATTTTGTGGGAATTGGTATGG |
|  | *gki*-2 | ACCATTAAAGCAAAATGATCGC |
| *aroE* | *aroE* -1 | TGGAAAACTTTACGGAGACAGC |
|  | *aroE* -2 | GTCCTGTCCATTGTTCAAAAGC |
| *xpt* | *xpt*-1 | AAAATGATGGCCGTGTATTAGG |
|  | *xpt*-2 | AACGTCACCGTTCCTTCACTTA |
| *yqiL* | *yqiL* -1 | CAGCTTAAGTCAAGTAAGTGCCG |
|  | *yqiL* -2 | GAATATCCCTTCTGCTTGTGCT |

ping genes in *Enterococcus faecalis*

| Fragment size (bp) | References |
| --- | --- |
| 530 | Ruiz-Garbajosa P et al. 2006 |
| 395 | Ruiz-Garbajosa P et al. 2006 |
| 583 | Ruiz-Garbajosa P et al. 2006 |
| 438 | Ruiz-Garbajosa P et al. 2006 |
| 459 | Ruiz-Garbajosa P et al. 2006 |
| 456 | Ruiz-Garbajosa P et al. 2006 |
| 436 | Ruiz-Garbajosa P et al. 2006 |


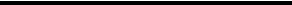


PCR Conditions


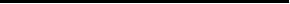


Initial denaturation: 94°C for 5 min

30 cycles:

- Denaturation: 94°C for 30 s

- Annealing: 52°C for 30 s

- Extension: 72°C for 1 min

Final extension: 72°C for 7 min


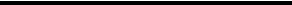

Supplement: Table S1 — The primers used for MLST typing of housekeeping genes in Enterococcus faecalis. [file spectrum.01217-24-s0001.docx]
