# Supplementary material for: Antimicrobial resistance, virulence gene profiles, and molecular epidemiology of enterococcal isolates from patients with urinary tract infections in Shanghai, China
Source: Microbiol Spectr. 2024 Nov 29;13(1):e01217-24. doi: 10.1128/spectrum.01217-24 (PMC11705914; doi:10.1128/spectrum.01217-24)

**Table S2**: The Primers used for MLST typing of housekeeping genes in *Enterococcus faecium*

| Gene | Primer Name | Primer Sequence (5' → 3') |
| --- | --- | --- |
| *adk* | adk-1 | TATGAACCTCATTTTAATGGG |
|  | adk-2 | GTTGACTGCCAAACGATTTT |
| *atpA* | atpA-1 | CGGTTCATACGGAATGGCACA |
|  | atpA-2 | AAGTTCACGATAAGCCACGG |
| *ddl* | ddl-1 | GAGACATTGAATATGCCTTATG |
|  | ddl-2 | AAAAAGAAATCGCACCG |
| *gdh* | gdh-1 | GGCGCACTAAAAGATATGGT |
|  | gdh-2 | CCAAGATTGGGCAACTTCGTCCCA |
| *gyd* | gyd-1 | CAAACTGCTTAGCTCCAATGGC |
|  | gyd-2 | CATTTCGTTGTCATACCAAGC |
| *purK* | purK-1 | GCAGATTGGCACATTGAAAGT |
|  | purK-2 | TACATAAATCCCGCCTGTTTCT |
| *pstS* | pstS-1 | TTGAGCCAAGTCGAAGCTGGAG |
|  | pstS-2 | CGTGATCACGTTCTACTTCC |

genes in *Enterococcus faecium*

| Fragment size (bp) | References |
| --- | --- |
| 437 | Homan WL et al. 2002 |
| 556 | Homan WL et al. 2002 |
| 465 | Homan WL et al. 2002 |
| 530 | Homan WL et al. 2002 |
| 395 | Homan WL et al. 2002 |
| 492 | Homan WL et al. 2002 |
| 583 | Homan WL et al. 2002 |


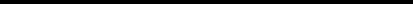


PCR Conditions


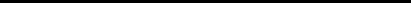


Initial denaturation: 94°C for 3 min

35 cycles:

- Denaturation: 94°C for 30 s

- Annealing: 50°C for 30 s

- Extension: 72°C for 30 s

Final extension: 72°C for 5 min


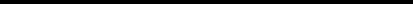

Supplement: Table S2 — The primers used for MLST typing of housekeeping genes in Enterococcus faecium. [file spectrum.01217-24-s0002.docx]
